# Differentiation of Palmoplantar Psoriasis, Palmoplantar Pustulosis and Hyperkeratotic Palmoplantar Eczema Using Proteomic Analysis of Tape Strip Samples

**DOI:** 10.1111/exd.70318

**Published:** 2026-07-10

**Authors:** Mila B. Johansen, Sule Altintas, Mariana Bronze, Anders Woetmann, Claus Zachariae, Charlotte Näslund‐Koch, Marianne B. Løvendorf, Lone Skov

**Affiliations:** ^1^ Department of Dermatology and Allergy Copenhagen University Hospital—Herlev and Gentofte Copenhagen Denmark; ^2^ Department of Clinical Medicine, Faculty of Health and Sciences University of Copenhagen Copenhagen Denmark; ^3^ Department of Immunology and Microbiology, Faculty of Health and Medical Sciences, The Leo Foundation Skin Immunology Research Center University of Copenhagen Copenhagen Denmark

**Keywords:** hyperkeratosis palmaris et plantaris, proteomics, psoriasis, pustulosis of palms and soles, skin diseases

## Abstract

Palmoplantar inflammatory dermatoses, including hyperkeratotic palmoplantar eczema (HPE), palmoplantar psoriasis (PP) and palmoplantar pustulosis (PPP), present overlapping clinical features that complicate diagnosis and limit targeted therapy. This prospective observational study evaluated whether minimally invasive tape strip sampling, combined with targeted proteomics, can distinguish these conditions at a molecular level. Adults with HPE (*n* = 14), PP (*n* = 10), and PPP (*n* = 12) were enrolled, and tape strips from esional and non‐lesional skin were analysed using the Olink Reveal panel (1034 proteins) with validation by MSD multiplex immunoassays. Differentially expressed proteins, pathway enrichment analysis, and protein–protein interaction analyses were conducted across the three diseases, and longitudinal analyses were conducted in a subset of patients with PP. Protein expression patterns showed partial separation of PPP from PP and HPE, whereas PP and HPE overlapped. Lesional samples across all three diagnoses exhibited increased expression of inflammatory proteins compared with non‐lesional skin. PPP showed a distinct molecular profile from PP and HPE, with enriched pathways related to neutrophil degranulation, innate immune activation, and Th17‐associated signalling networks centred on IL‐17A and CXCL8. No proteins were significantly differentially expressed between PP and HPE lesions. PP and HPE showed overlapping interferon‐associated chemokines CXCL10‐11, consistent with a shared inflammatory module. Inflammatory protein levels decline with clinical improvement in longitudinal PP samples. Overall, tape strip‐based targeted proteomics is a feasible approach for palmoplantar diseases, revealing a distinct inflammatory signature that differentiates PPP from PP and HPE, although discrimination between PP and HPE remains limited.

## Graphical abstract

Tape strip‐based targeted proteomics reveals specific protein patterns in PPP compared with PP and HPE. The findings demonstrate the feasibility of tape strip proteomics to differentiate PPP from PP and HPE.

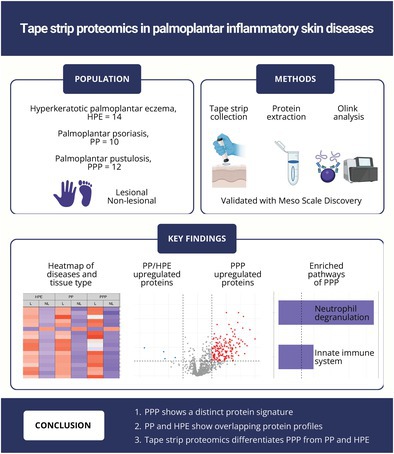

## 1 Introduction

Palmoplantar involvement in inflammatory skin diseases, including hyperkeratotic palmoplantar eczema (HPE), palmoplantar psoriasis (PP) and palmoplantar pustulosis (PPP), presents diagnostic and therapeutic challenges [1, 2, 3, 4]. Despite limited body surface involvement, palmoplantar diseases have a substantial impact on the quality of life of the patients [1, 5, 6, 7], and are classified as a high‐impact site [8, 9], leading to a higher disease severity classification based solely on localisation [10].

The clinical diagnosis of PP is based on acral localisation, which typically presents as well‐demarcated, erythematous, hyperkeratotic plaques, sometimes accompanied by psoriasis vulgaris lesions on other body sites [1, 11, 12, 13]. In PPP, an erythematous, desquamated base on the palms or soles, with sterile pustules in certain stages, is observed [14]. An important differential diagnosis of PP and PPP is HPE [1], which presents as chronic hyperkeratotic palmoplantar lesions, often centrally located on the acral skin without vesicles or visible inflammation [15].

Although histology and dermoscopy can assist in the diagnosis, differentiation remains difficult due to anatomical constraints and overlapping clinical features [11, 12, 13, 16, 17, 18]. Previous studies have explored the immunological differences among HPE, PP and PPP, primarily using immunohistochemistry and transcriptomics [12, 19, 20, 21, 22, 23, 24, 25]. Increased interleukin (IL)‐17 expression has been reported in PPP compared with PP [19, 22, 23]. IL‐36γ appears to be more strongly expressed in PPP [22, 23], but also appears in PP compared with HPE [12, 20], while IL‐23 is expressed in all three diseases [24]. IL‐8 is upregulated in PPP [21, 22].

Given the diagnostic uncertainty and the practical limitations of biopsy in palmoplantar disease, there is a need for alternative, non‐invasive diagnostic approaches. Tape stripping provides a minimally invasive method for sampling the stratum corneum and has been used in molecular studies of different inflammatory skin diseases [26, 27, 28, 29, 30, 31, 32, 33, 34]. Proteomic analysis enables characterization of disease‐specific molecular profiles, but has been sparsely applied to palmoplantar conditions [33, 35, 36, 37, 38, 39]. In this study, we use tape strip sampling combined with targeted proteomic analysis to investigate whether HPE, PP and PPP can be distinguished at the molecular level.

## 2 Materials and Methods

### 2.1 Study Design and Population

This is a prospective cross‐sectional observational study with a longitudinal sub‐study. Participants ≥ 18 years with HPE, PP and PPP were recruited from February 2022 to May 2025 from the Department of Dermatology and Allergy, Herlev and Gentofte Hospital, Denmark, as part of the Copenhagen translational skin immunology biobank and research program (BIOSKIN) [40]. Before inclusion, a clinical diagnosis of palmoplantar disease was made by a dermatologist. In cases of diagnostic uncertainty, a second opinion was obtained from a senior consultant in dermatology. A complete skin examination was performed on all patients. Clinically, PP was defined by the presence of sharply demarcated, erythematous plaques on the palmoplantar areas. Concomitant or a history of psoriasis vulgaris lesions at other anatomical sites supported the diagnosis of PP but was not a requirement. In PPP, lesions were clinically characterized by a palmoplantar erythematous, desquamative base and yellow pustules or residual brown macules consistent with resolving pustules. If a few pustules were present, a prior history of pustules documented in medical records or photographs supported the diagnosis. The clinical diagnosis of HPE was based on chronic hyperkeratotic lesions primarily affecting the central acral skin, without clinical signs of vesiculation, current extra‐palmoplantar psoriasis vulgaris lesions, or a known psoriasis diagnosis. Inclusion criteria required that patients had not received ultraviolet (UV) light therapy or systemic treatment within the past month and had not received advanced immunological systemic treatment within the past 3 months. A minority of patients were receiving topical treatment at the time of enrolment (Table S1). From six patients with PP, tape strips were also collected at a follow‐up visit 6–12 months after the baseline visit (Table S2).

### 2.2 Sample Collection

Tape strips were collected from lesional skin (L, hand/ft) and non‐lesional skin (NL, from different anatomical places) of all patients. Non‐lesional skin was defined as unaffected skin located ≥ 10 cm from any active lesion. At a follow‐up visit, six patients with PP were evaluated. Healed skin was defined as previously lesional skin that had achieved complete clinical resolution with no visible signs of inflammation. At each site, eight consecutive tape strips (22‐mm D100 D‐Squame adhesive discs; Clinical & Derm) were applied for 10 s at a standardized pressure of 225 g/cm^2^ using a D500 D‐Squame pressure instrument. Tape strips were removed using forceps, placed adhesive‐side inward into 2 mL microtubes (Hounisen), and stored at −80°C. Whole blood samples were analysed for the HLA‐C*06:02 gene variant at the Department of Clinical Biochemistry, Herlev and Gentofte Hospital, Denmark.

### 2.3 Olink Analysis

Four tape strips (numbers 1, 3, 5 and 7) were pooled for proteomic analysis at the Danish Technical University (DTU), Department of Health Technology, Center for Diagnostics, Denmark. A total of 650 μL lysis buffer (0.2% Triton X‐100 in PBS with Complete Mini Protease Inhibitor Cocktail; Roche) was added to the first tape strip. The sample was briefly vortexed, centrifuged at 4°C for 30 s, and sonicated for 30 s on/30 s off/30 s on (50% amplitude) using an ultrasonic processor equipped with a cup‐horn attachment (Fisher Scientific, Pittsburgh, PA, USA; model FB505). The tape strip was then removed, and the procedure was repeated sequentially for each of the remaining tape strips using the same lysis buffer (Figure S1). A Pierce BCA protein assay (Thermo Fisher Scientific, Waltham, MA, USA) was used to determine the protein concentrations. The Olink Reveal panel (Olink Proteomics AB, Uppsala, Sweden), containing 1034 proteins, was used to quantify the protein extracts from the tapes. The output was a log_2_‐scaled quantification of the relative protein abundance (NPX value). Interplate control (IPC) normalization was used to correct for interplate variation according to the manufacturer's protocol.

### 2.4 Meso Scale Discovery (MSD) Analysis

Selected proteins were quantified in the same protein extracts used for the Olink analyses using the MSD platform (Meso Scale Diagnostics LLC, Rockville, MD), including sensitive U‐PLEX (IL‐23, CXCL10/IP‐10) and ultra‐sensitive S‐PLEX (IL‐22, TNF‐α, IL‐17A) human assays. Six samples (lesional = 3, non‐lesional = 3) from patients with HPE with the lowest protein concentrations were excluded due to limited plate capacity. Calibrators were run in duplicate, and plates were immediately read on a MESO QuickPlex SQ120 instrument (Meso Scale Diagnostics, Rockville, MD, USA). The log signal of each calibrator was plotted versus the concentration of each calibrator by MSD Workbench 4.0 to create a standard curve to infer protein concentrations in the samples. The lower limit of detection (LOD) was defined as 2.5 standard deviations above the zero calibrator. The lower limit of quantification (LLOQ) was obtained from assay‐specific information provided in MSD Workbench, and concentrations below the LLOQ were considered not reliably quantifiable. Technical differences between assays are summarized in Table S3.

### 2.5 Statistical Analysis

Statistical analyses were performed in R (version 4.5.2) [41]. Baseline characteristics are presented as medians and interquartile ranges (IQR) for continuous variables and as frequencies and percentages for categorical variables. Between‐group differences were assessed using the Kruskal–Wallis test for continuous variables and Fisher's exact test for categorical variables. Pairwise comparisons were performed using the Mann–Whitney *U* test.

All cross‐sectional analyses followed a stepwise framework comprising descriptive comparisons, exploratory multivariate analyses, differential protein expression testing, functional interpretation and network‐based analyses. Normalized protein expression (NPX) values were first compared across the three palmoplantar disease groups (HPE, PP and PPP) at a single visit to assess global proteomic patterns [42]. Principal component analysis was used to visualize clustering of samples. Variance partitioning was performed to estimate the proportion of total variance. Differentially expressed proteins (DEPs) between disease groups and between lesional and non‐lesional samples were identified using linear models with empirical Bayes moderation implemented in the *limma* package [43]. Proteins with an absolute log_2_ fold change (|log_2_FC|) ≥ 1 and a Benjamini‐Hochberg false discovery rate (FDR)‐adjusted *p* < 0.05 were considered significantly differentially expressed. Heatmaps were performed using *ComplexHeatmap* in R [44]. Volcano plots were generated to visualize differential expression across disease comparisons in lesional samples (PP‐HPE, PPP‐HPE and PPP‐PP) [45]. Functional interpretation of DEPs in lesional skin was performed using *ReactomeRA* over‐representation analysis [46]. Pathways with FDR‐adjusted *p* < 0.05 were considered significant and ranked by adjusted *p*‐value. Protein–protein interaction networks were constructed for the top 50 DEPs in PPP‐HPE and PPP‐PP comparisons using STRING (version 12.0) [47]. Networks included the query proteins and high‐confidence interactions (combined score ≥ 0.7), with an inflation parameter of 2.5 to control clustering tightness.

A longitudinal sub‐analysis was subsequently performed for the subset of six patients with PP with follow‐up samples from healed or lesional skin. For each protein, paired NPX differences were calculated within patients.

## 3 Results

### 3.1 Participant Characteristics and Sample Quality Assessment

This cross‐sectional study comprised 14 patients with HPE, 10 patients with PP and 12 patients with PPP. Baseline characteristics are summarized in Table 1 and Tables S1 and S2. All samples fulfilled the predefined quality control criteria based on internal assay controls and plate control performance (Table 2). Protein concentration distributions (Figure S2) were generally higher in lesional than in non‐lesional samples, with the highest levels observed in PPP. All samples were retained for downstream analyses.

**TABLE 1:** Baseline characteristics of hyperkeratotic palmoplantar eczema (HPE), palmoplantar psoriasis (PP) and palmoplantar pustulosis (PPP).

Baseline data	Patients with HPE (*n* = 14)	Patients with PP (*n* = 10)	Patients with PPP (*n* = 12)	*p*
Age, years, median (IQR)^b^	56.5 (51.3–65.0)	63 (47.8–72.8)	62 (42.5–66.5)	0.697^c^
Female, *n* (%)^b^	8 (57.1)	7 (70.0)	9 (75.0)	0.672^d^
BMI, kg/m^2^, median (IQR)^b^	27.8 (25.7–32.3)	25.8 (25.5–30.7)	27.7 (23.9–30.3)	0.767^c^
Age at diagnosis, years, median (IQR)^a^	53 (31.0–56.0)	38.5 (19.3–67.0)	27.5 (23.0–52.0)	0.757^c^
Disease duration, years, median (IQR)^a^	4 (1.0–12.0)	14.5 (9.0–24.3)	11.5 (6.8–20.7)	0.308^c^
Genetic disposition for psoriasis, *n* (%)^a^				
Yes	6 (42.9)	6 (60.0)	7 (58.3)	0.217^d^
No	8 (57.1)	4 (40.0)	4 (33.3)	
Unknown	0 (0)	0 (0)	1 (8.3)	
Smoking, *n* (%)^a^				
Never	5 (35.7)	3 (30.0)	0 (0)	0.047^d^
Former	2 (14.3)	5 (50.0)	5 (41.7)	
Current	7 (50.0)	2 (20.0)	7 (58.3)	
DLQI, total, median (IQR)^a^	9.0 (6.5–12.0)	4.5 (4.0–7.5)	6.5 (3.5–7.3)	0.305^c^

*Note:* Median with IQR (25th–75th percentiles) for non‐normally distributed continuous variables. Number and frequency as percentages for categorical variables. A *p*‐value below 0.05 was considered significant.

Abbreviations: %, percent; BMI, body mass index; DLQI, dermatology life quality index; HPE, hyperkeratotic palmoplantar eczema; IQR, interquartile range; *n*, number; PP, palmoplantar psoriasis; PPP, palmoplantar pustulosis.

^a^ Patient‐reported questionnaires.

^b^ Health‐care professional examination and questionnaires.

^c^ The Kruskal–Wallis test is used for numeric data, given the presence of unpaired non‐distributed data from three groups.

^d^ Fisher's Exact test is used for categorical data, given the presence of small sample sizes in each disease group.

**TABLE 2:** Overview of Olink Reveal data quality.

	All (*n* = 36)	HPE (*n* = 14)		PP (*n* = 10)		PPP (*n* = 12)	
		L	NL	L	NL	L	NL
Samples for all protein assays, *n*	76 516	15 510	15 510	10 340	10 340	12 408	12 408
Passed quality control, *n* (%)	76 516 (100)	15 510 (100)	15 510 (100)	10 340 (100)	10 340 (100)	12 408 (100)	12 408 (100)
NPX values above lower LOD, *n* (%)^a^	67 653 (88.4)	13 835 (89.2)	13 547 (87.3)	9250 (89.4)	9027 (87.3)	11 216 (90.4)	10 778 (86.9)

Abbreviations: %, percent; HPE, hyperkeratotic palmoplantar eczema; L, lesional; *n*, number; NL, non‐lesional; PP, palmoplantar psoriasis; PPP, palmoplantar pustulosis.

^a^ LOD: Limit of detection is the minimum level of an individual protein assay that can be measured. Predetermined fixed LOD values for Olink Reveal have been used.

### 3.2 Lesional and Non‐Lesional Skin Show Distinct Proteomic Profiles With a Pronounced Inflammatory Signature in PPP

**FIGURE 1:**
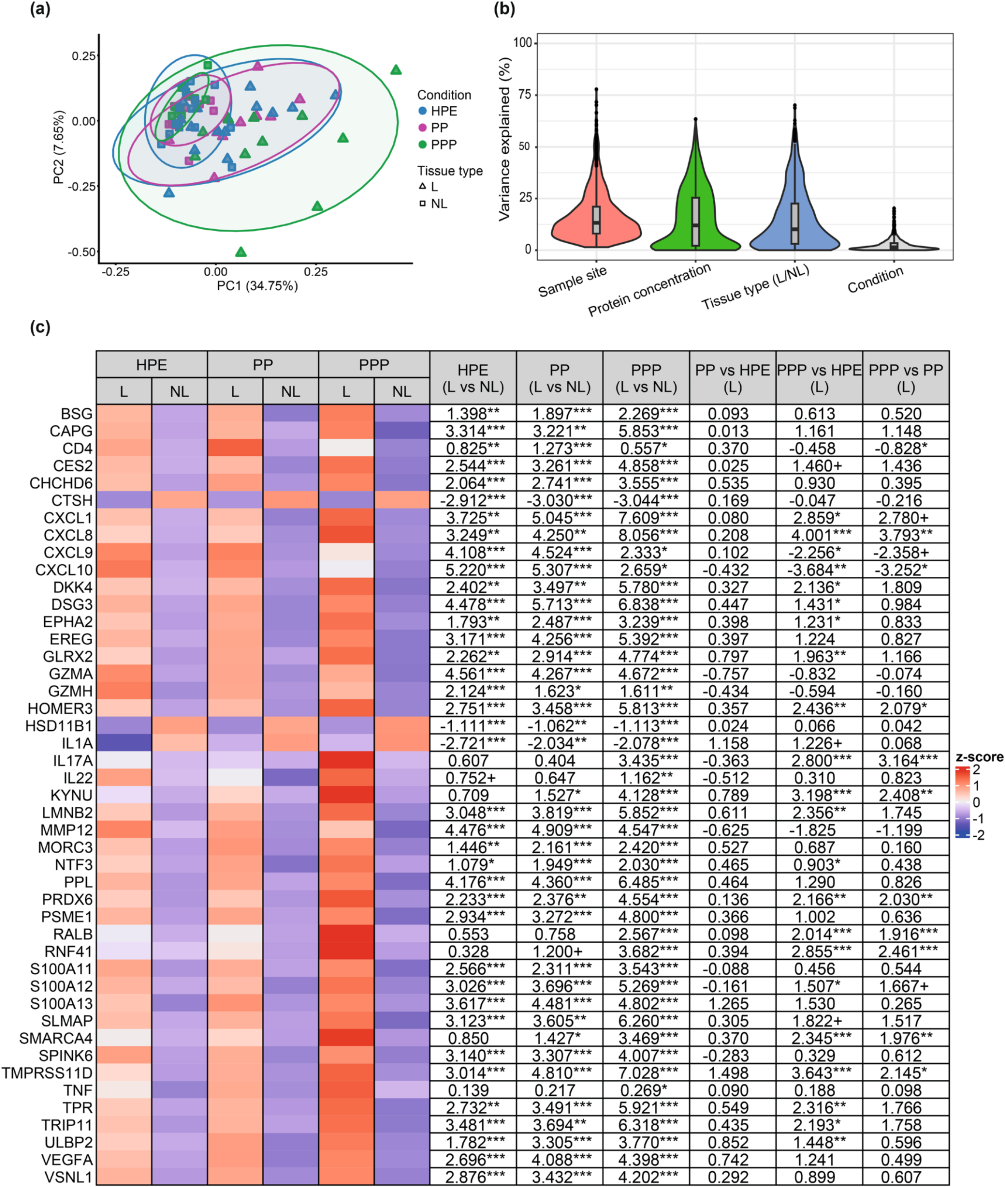
(a–c) Proteomic profiling reveals distinct clustering of lesional samples and a more distinct profile for PPP compared with overlapping PP and HPE profiles. (a) Principal component analysis of protein expression profiles in hyperkeratotic palmoplantar eczema (HPE), palmoplantar psoriasis (PP) and palmoplantar pustulosis (PPP). Each point represents one sample, coloured by disease condition and shaped according to tissue type (L: Lesional, triangle; NL: Non‐lesional, square). Principal component 1 (PC1) explains 34.75% of the total variance, and PC2 explains 7.65%. The shaded ellipses indicate the regions for each disease group. (b) Variance partitioning analysis showing the proportion of explained variance (*y*‐axis), attributable to sample site, protein concentration, tissue type (L/NL), and condition (*x*‐axis). Each violin depicts the distribution of variance contributions across all proteins, with the embedded boxplot indicating the median and interquartile range. (c) Heatmap showing disease and tissue‐type comparisons based on the top 20 significantly different proteins for each of the three diseases (HPE, PP and PPP) and lesional (L) and non‐lesional (NL) samples. Values are standardized as *z*‐scores, meaning expression is shown relative to each protein's mean across all samples. Red = higher expression, blue = lower expression.

Following confirmation of data quality, the global proteomic structure was examined across disease groups and tissue types (lesional/non‐lesional). Principal component analysis showed broader dispersion and partial separation of PPP samples, consistent with a more distinct molecular signature, whereas PP and HPE exhibited overlapping proteomic profiles (Figure 1a). Variance partitioning revealed that sample site, protein concentration, and tissue type explained a greater proportion of variance than disease condition (Figure 1b). Sex, age and smoking contributed minimally (data not shown).

To further examine differences between lesional and non‐lesional skin, a combined heatmap was generated that included the top 20 differentially expressed proteins (DEPs) identified for each disease. In addition, three proteins of interest (IL‐17A, IL‐22 and TNF‐α) were selected and added to the heatmap (Figure 1c). Across all three conditions, lesional samples showed a consistent shift towards increased expression of inflammatory proteins compared with non‐lesional skin. Among the 44 proteins included in the heatmap, 40 were upregulated in lesional skin, whereas four proteins (CTSH, HSD11B1, IL‐1A and SCPEP1) showed higher expression in non‐lesional samples. This pattern resulted in a clear separation between lesional and non‐lesional samples across diseases. Within lesional samples, disease‐specific differences were less pronounced. However, PPP demonstrated a greater number of strongly upregulated inflammatory mediators compared with PP and HPE. Because lesion status accounted for a substantial proportion of global proteomic variation, subsequent analyses were restricted to lesional samples to investigate disease‐specific molecular differences.

### 3.3 Lesional Proteomic Analysis Distinguishes PPP by a Neutrophil‐Driven Inflammatory Signature

**FIGURE 2:**
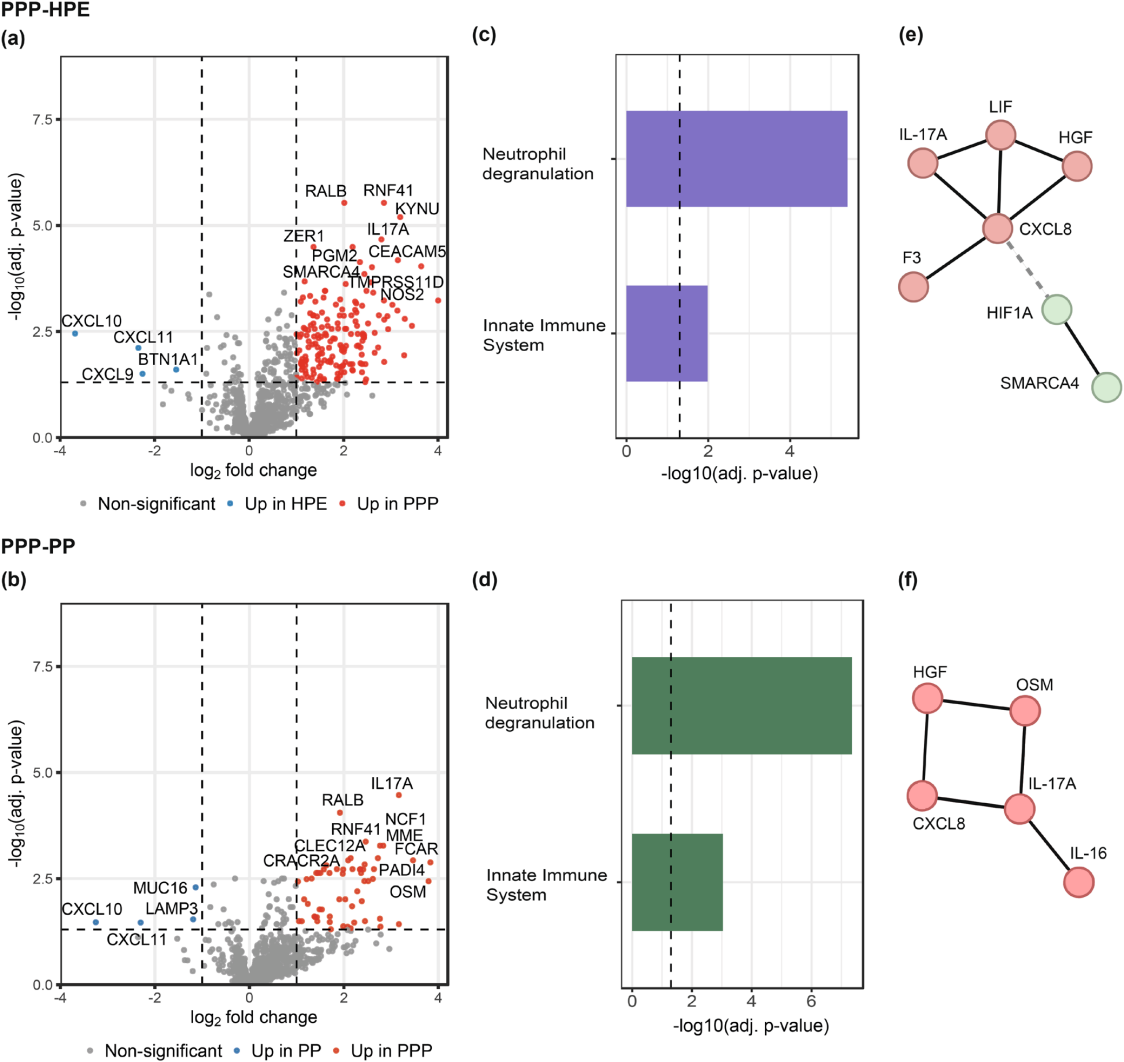
(a–f) PPP presents a distinct inflammatory proteomic signature and pathways in lesional samples, while PP and HPE share similar signatures and pathways. (a, b) Volcano plot showing differential protein expression in lesional skin between disease groups. The *x*‐axis represents log_2_ fold change, the *y*‐axis represents −log_10_ adjusted *p*‐value. Vertical dashed lines indicate the fold‐change threshold, and the horizontal dashed line indicates the significance threshold (adjusted *p*‐value). Each dot represents one protein; grey dots denote non‐significant proteins, while coloured dots indicate significantly differentially expressed proteins in the indicated condition (red in the first‐mentioned disease, blue in the second‐mentioned disease). The top 10 selected significant proteins are labelled by their assay names. (c, d) Reactome pathway over‐representation analysis (ORA) of differentially expressed proteins in lesional skin comparisons. Bar plots show significantly enriched pathways identified using all differentially expressed proteins for PPP‐HPE and PPP‐PP comparisons. The *x*‐axis displays −log_10_ (FDR‐adjusted *p*‐value), and the dashed vertical line indicates the significance threshold (FDR = 0.05). Enrichment was detected only in contrasts involving PPP. No pathway enrichment could be robustly inferred for PP or HPE due to the small number of differentially expressed proteins. (e, f) STRING protein–protein interaction (PPI) clustering network of the top 50 most significant DEPs of the comparisons PPP‐HPE and PPP‐PP, mainly composed of DEPs highest in PPP. Edge thickness reflects the combined confidence score. Nodes are coloured according to cluster assignment. The dotted edges show a functional association between proteins. *PPP‐HPE*: Cluster 1 (orange) represents positive regulation of positive chemotaxis (CXCL8, IL‐17A, LIF, HGF, F3). Cluster 2 (green) comprises HIF1A and SMARCA4. *PPP‐PP*: Cluster 1 (red) represents induction of positive chemotaxis (CXCL8, IL‐17A, HGF, OSM, IL‐16).

Pairwise comparisons of lesional skin between disease groups showed that PPP differed significantly from PP and HPE, with 170 and 63 DEPs identified in the PPP‐HPE and PPP‐PP comparisons, respectively, most of which were upregulated in PPP (Figure 2a,b). In contrast, no proteins met the significance criteria in the PP‐HPE comparison, indicating similar proteomic profiles (Figure S3). Consistently upregulated proteins in PPP—including RALB, RNF41, IL‐17A, FCAR, NOS2, ADAM8 and SMARCA4—are associated with inflammatory signalling, IL‐17‐driven neutrophil responses, the innate immune response, and transcriptional regulation of inflammatory genes. PP and HPE were characterized by upregulated interferon‐γ‐associated chemokines CXCL10 and CXCL11.

To further characterize these disease‐specific differences, pathway enrichment analysis was performed (Figure 2c,d; Table S4). Pathway enrichment was observed only in comparisons involving PPP, reflecting the larger number of DEPs identified in PPP. In contrast, PP and HPE showed limited signal, with only four upregulated proteins detected in each group. Proteins distinguishing PPP from the other disease groups were enriched for neutrophil degranulation and innate immune system pathways, supported by markers including ADAM8, CXCL1, CLEC12A and NOS2. Protein–protein interaction network analysis further supported these findings (Figure 2e,f). In the PPP‐HPE comparison, an inflammatory core centred on IL‐17A and CXCL8 connected to cytokine, stress and metabolic regulators (LIF, HGF, HIF1A) as well as chromatin‐dependent regulators of transcription (SMARCA4) was consistent with coordinated Th17 signalling and neutrophil recruitment. Similarly, the PPP‐PP comparison showed a compact IL‐17A‐centered module linked to OSM, IL‐16 and CXCL8‐HGF signalling. Collectively, these analyses indicate that PPP is characterized by a Th17‐neutrophil inflammatory network.

### 3.4 MSD Validation Confirms Detection Patterns of Selected Inflammatory Proteins

**FIGURE 3:**
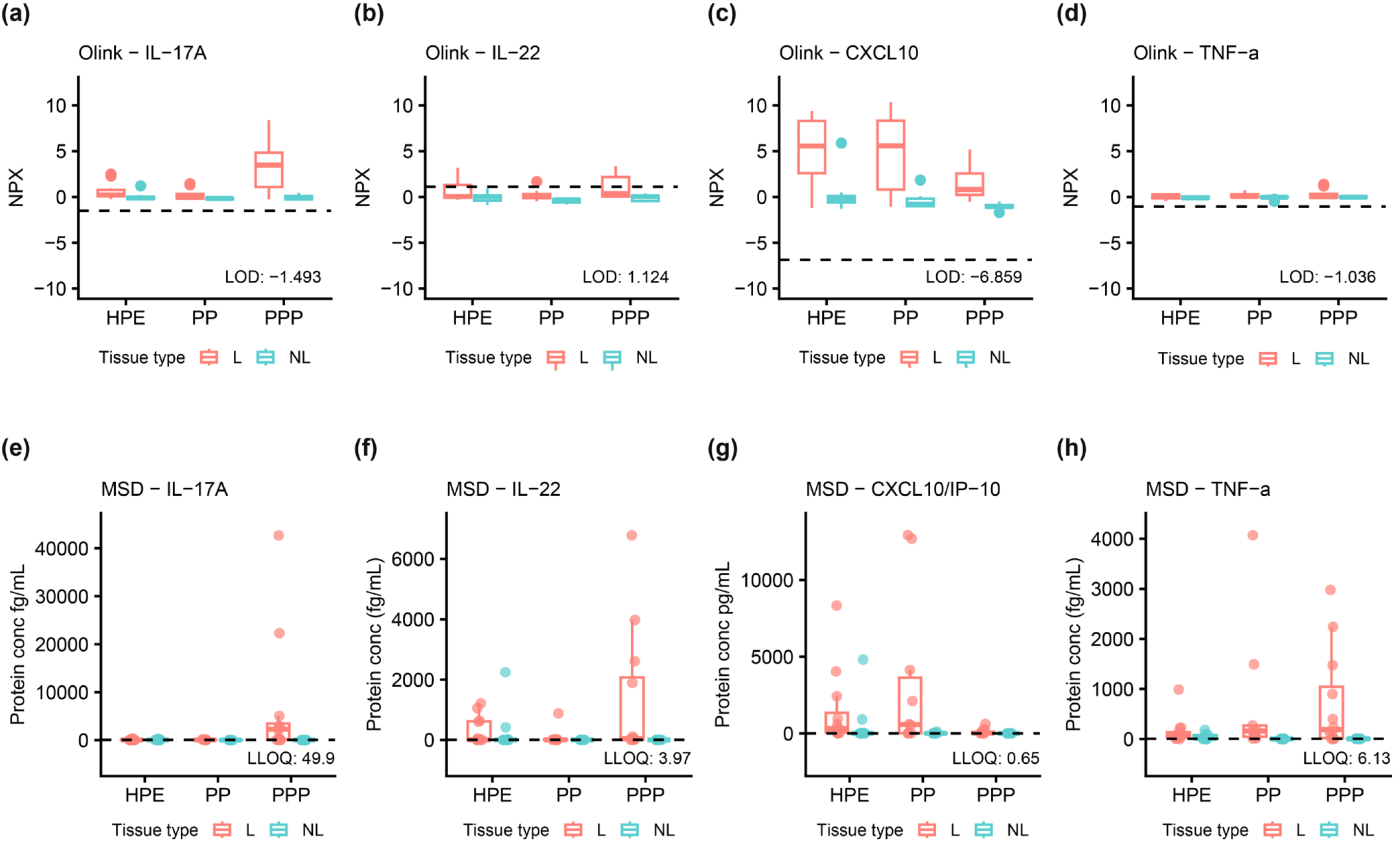
(a–h) Comparable protein sensitivity of Olink and MSD platforms across four selected proteins, with modest assay‐specific differences for TNF‐α and IL‐22. Side‐by‐side boxplots display protein levels measured by (a–d) Olink (NPX) and (e–h) MSD (fg/mL or pg/mL) across disease groups (HPE, PP, PPP). Lesional skin: L, red bars; non‐lesional skin: NL, turquoise bars. Dotted lines indicate limits of quantification (LOD) or lower limit of quantification (LLOQ). Olink data are reported as relative normalized protein expression (NPX). MSD data provides absolute concentration measurements based on standard curve interpolation with assay‐specific LLOQs.

We next validated the detection patterns of four proteins identified by Olink Reveal, along with IL‐23, using the MSD platform (Figure 3a–h). IL‐17A and CXCL10/IP‐10 showed concordant patterns with Olink, while TNFα and IL‐22 demonstrated higher detectability with MSD. IL‐23 was undetectable in all samples (data not shown). Boxplots of selected proteins from the Olink dataset included in downstream analysis are shown in Figure S4, including NPX values and the corresponding LOD.

### 3.5 Inflammatory Protein Levels Decline With Clinical Improvement in Longitudinal PP Samples

**FIGURE 4:**
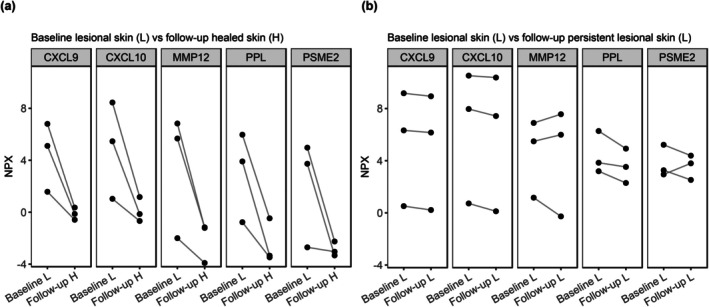
(a, b) Inflammatory protein levels decline with clinical improvement in longitudinal samples from patients with palmoplantar psoriasis (PP). Data from six patients with PP at baseline and at follow‐up. Three patients healed during follow‐up (a), and three patients had no change in disease activity (b). Each dot represents an individual measurement, and each line connects paired samples from the same patient, illustrating within‐subject longitudinal changes. (a) Paired NPX trajectories for proteins distinguishing baseline lesional (L) skin and healed (H) skin at follow‐up visit (L–H, *n* = 3). All five proteins across three patients indicate downregulation with lesion resolution. (b) Paired NPX trajectories for proteins distinguishing proteins from L–H comparison but based on patients with samples from baseline lesional (L) skin and persistent lesional (L) skin at follow‐up (L–L, *n* = 3).

To assess whether inflammatory protein levels change with clinical improvement, paired longitudinal samples from six patients with PP were analysed at baseline and follow‐up. Three patients showed healed lesions at follow‐up, whereas three had persistent lesions. The five proteins showing the most consistent directional changes between baseline lesional samples and follow‐up healed samples (*n* = 3) were CXCL9, CXCL10, MMP12, PPL and PSME2 (Figure 4a; Table S5). In patients with persistent lesions at follow‐up (*n* = 3), the expression of these proteins remained comparable to baseline or changed only modestly (Figure 4b, Table S5).

## 4 Discussion

The palmoplantar inflammatory skin diseases HPE, PP and PPP present a considerable diagnostic challenge due to the overlap in clinical features [4, 11, 13, 14]. Tape strip proteomics offers a practical, non‐invasive approach in acral sites where biopsies are challenging [48]. In this study, targeted proteomic profiling of tape strip samples was used to assess whether HPE, PP and PPP could be distinguished at the molecular level in the stratum corneum. Four key findings were identified. First, lesional skin samples contributed mostly to global proteomic variation, particularly in PPP. Second, PPP showed a proteomic distinction from both PP and HPE. Third, PP and HPE showed overlapping lesional modules; and lastly, in a subset of six patients with PP, five selected proteins showed declining changes over time in inflammatory proteins.

In this study, PPP was characterized by a coherent inflammatory module centred on IL‐17A and neutrophil mediators, with enrichment of neutrophil degranulation and innate immune pathways. Proteins including IL‐17A, ADAM8, CXCL8, CLEC12A, FCAR and NOS2 sup this profile. Additional proteins upregulated in PPP, such as RALB [49], RNF41 [50] and SMARCA4 [51], suggest broader regulation of inflammatory signalling and transcriptional control consistent with activation of a coordinated Th17‐neutrophil axis. These findings align with previous evidence implicating Th17‐driven inflammation and neutrophil recruitment in PPP [19, 23, 25, 52, 53, 54, 55, 56] and with reports suggesting that IL‐17 may also be produced by neutrophils [53]. Immune plasticity in PPP, including Th17‐to‐Th2 transitions [19, 54, 57], may contribute to stage‐dependent inflammatory variation in PPP and support the concept of a distinct inflammatory profile [52]. The IL‐17‐centred inflammatory network observed can support the rationale for IL‐17‐targeted therapies [53, 58]. However, IL‐17 inhibition may paradoxically induce PPP in some cases and with variable effectiveness [59, 60, 61]. This variability may reflect the broader inflammatory architecture suggested by our data and other studies [54, 56]. Consequently, therapeutic approaches targeting alternative pathways, including Th2‐directed therapies or broader inflammatory signalling via JAK inhibition, may be relevant [57, 62]. Despite the continuing debate on the classification and treatment of PPP [52, 63], PPP exhibits a specific proteomic profile at the stratum corneum level in our data.

In contrast, there were no coherent profiles or significant differences detected between PP and HPE in lesional skin. Both conditions were characterized by expression of interferon‐associated chemokines such as CXCL10 and CXCL11, suggesting shared inflammatory signalling pathways. Histopathological studies have similarly reported limited differences between PP and HPE [11, 12, 13].

Transcriptomic analyses have proposed IL‐17A as a potential discriminator between PP and HPE, whereas IFN‐γ was reported to be expressed at comparable levels in both conditions [64]. The IL‐17A distinction was not observed in the present study. Consistent with IFN‐γ pathway activity, the associated proteins CXCL10 and CXCL11 were detected in our dataset. Other cytokines previously implicated in disease differentiation, including IL‐13 and IL‐23 [24, 64], were detected at low levels. These findings may indicate that potential discriminatory signals are located deeper in the skin, which are not captured by tape stripping [32, 65, 66]. Lesion‐driven patterns have been reported in non‐invasive transcriptomic studies of psoriasis and eczema [29, 32, 33].

Consistent with the dominance of lesional signals, exploratory longitudinal analyses in a small subset of patients with PP showed declining changes over time in inflammatory proteins (CXCL9, CXCL10, MMP12, PPL and PSME2), suggesting potential markers of treatment response. These findings indicate that tape strip proteomics may capture dynamic inflammatory changes, consistent with transcriptomic and proteomics studies [67, 68]. CXCL9 and CXCL10 are IFN‐γ‐Th1‐associated chemokines, MMP12 is a matrix metalloproteinase that modifies the extracellular matrix, PPL is periplakin, which is an epidermal structural protein, and PSME2 has been described as an IFN‐γ regulator [69, 70, 71]. Although the limited number of longitudinal samples requires cautious interpretation.

The findings of this study should be considered according to the study design and methodological framework. Strengths include simultaneous analysis of three overlapping entities, within‐individual lesional and non‐lesional comparisons, integrated network analyses, and partial platform validation. Limitations comprise the targeted 1034‐protein panel, which restricts discovery beyond predefined proteins. Non‐lesional samples were from non‐acral sites within the same individuals, so it was not possible to isolate site‐specific biology [72]. The tape stripping method is restricted to the stratum corneum and upper epidermis, introducing depth‐related sampling bias and limiting insight into molecular signatures in deeper skin [32]. Moreover, subtle molecular differences in PP‐HPE may have remained undetected due to the modest sample size, combined with multiple‐testing correction to control for false discovery rate. Recruitment from a single centre restricts external generalizability, and the limited number of longitudinal samples reduces power to evaluate disease dynamics and treatment responsiveness.

## 5 Conclusion

In conclusion, PPP demonstrates a distinct Th17‐neutrophil‐associated proteomic signature detectable by non‐invasive tape strip sampling, whereas PP and HPE are not distinguishable using this targeted platform at the stratum corneum level. Tape strip‐based proteomics represents a feasible approach in palmoplantar disease, but its discriminatory capacity is condition‐dependent on PPP in this study. Further studies incorporating broader proteomic coverage and deeper tissue sampling are warranted to clarify the biological relationship between PP and HPE, as well as larger longitudinal studies investigating possible treatment response markers.

## Author Contributions

Conceptualization: M.B.J., L.S., M.B.L. data curation: M.B.J., S.A. formal analysis: M.B.J., S.A., M.B. funding acquisition: L.S. investigation: M.B.J., C.N.‐K., L.S., C.Z. methodology: M.B.J., S.A., L.S., M.B.L. project administration: L.S., C.Z. resources: L.S. supervision: L.S., M.B.L., A.W., C.Z. validation: M.B.J., M.B. visualization: M.B.J., S.A., L.S., M.B.L. writing – original draft preparation: M.B.J. writing – review and editing: M.B.J., L.S., M.B.L., S.A., M.B., C.N.‐K., C.Z., A.W.

## Funding

This work was supported by the LEO Foundation grant number (LF‐ST‐21‐500002) and the Copenhagen University Hospital—Herlev and Gentofte Hospital, Denmark.

## Ethics Statement

The project has been approved by the Scientific Ethical Committee of the Capital Region, Denmark (H‐21032986) and the Danish Data Protection Agency (P‐2021‐435). The study was conducted in accordance with the Helsinki Declaration.

## Consent

All participants provided informed, signed consent before enrolment.

## Conflicts of Interest

L.S. has been a paid speaker for AbbVie, Eli Lilly, Pfizer, Sanofi, Galderma and UCB, and has been a consultant or served on Advisory Boards with AbbVie, Janssen, Eli Lilly, LEO Pharma, UCB, Almirall, Boehringer Ingelheim, Bristol‐Myers Squibb, Takeda, Oruka Therapeutics and Sanofi. L.S. has received research and educational grants from UCB, Bristol‐Myers Squibb, Almirall, Sanofi and Janssen. C.Z. has been a consultant or served on Advisory Boards with Janssen Cilag, Novartis, Eli Lilly, LEO Pharma, UCB, Almirall and Takeda. C.Z. has been a paid speaker for UCB and LEO Pharma. C.N.‐K. has received honoraria as a consultant and/or speaker and a travel grant from LEO Pharma and UCB. The remaining authors declare no conflicts of interest.

## Supporting information


**Table S1:** Baseline characteristics of hyperkeratotic palmoplantar eczema (HPE), palmoplantar psoriasis (PP) and palmoplantar pustulosis (PPP).
**Table S2:** Participant demographics, disease severity and treatment characteristics for longitudinal data of palmoplantar psoriasis (PP).
**Table S3:** Key summary of variations in MSD S‐PLEX and U‐PLEX.
**Table S4:** Proteins in Reactome pathways.
**Table S5:** Mean NPX and changes from baseline to follow‐up for the top five proteins in palmoplantar psoriasis (PP).
**Figure S1:** Workflow of tape strip sampling, protein extraction, Olink Reveal analysis, next‐generation sequencing (NGS) and data analysis.
**Figure S2:** Protein concentration was highest in lesional samples and in palmoplantar pustulosis (PPP).
**Figure S3:** Palmoplantar psoriasis (PP) and hyperkeratotic palmoplantar eczema (HPE) present similar proteomic signatures.
**Figure S4:** Boxplots from selected proteins from the Olink Reveal panel.

## Data Availability

The data that support the findings of this study are available from the corresponding author upon reasonable request.
