# Supplementary material for: Differentiation of Palmoplantar Psoriasis, Palmoplantar Pustulosis and Hyperkeratotic Palmoplantar Eczema Using Proteomic Analysis of Tape Strip Samples
Source: Exp Dermatol. 2026 Jul 10;35(7):e70318. doi: 10.1111/exd.70318 (PMC13352295; doi:10.1111/exd.70318)

**Overview of supplementary material**

| **Overview of tables** | |
| --- | --- |
| **Table S1** | Baseline characteristics of hyperkeratotic palmoplantar eczema (HPE), palmoplantar psoriasis (PP), and palmoplantar pustulosis (PPP) |
| **Table S2** | Participant demographics, disease severity, and treatment characteristics for longitudinal data of palmoplantar psoriasis (PP) |
| **Table S3** | Key summary of variations in MSD S-PLEX and U-PLEX |
| **Table S4** | Proteins in Reactome pathways |
| **Table S5** | Mean NPX and changes from baseline to follow-up for the top five proteins in palmoplantar psoriasis (PP) |
| **Overview of figures** | |
| **Figure S1** | Workflow of tape strip sampling, protein extraction, Olink Reveal analysis, next-generation sequencing (NGS), and data analysis |
| **Figure S2** | Protein concentration was highest in lesional samples and in palmoplantar pustulosis (PPP) |
| **Figure S3** | Palmoplantar psoriasis (PP) and hyperkeratotic palmoplantar eczema (HPE) present similar proteomic signatures |
| **Figure S4** | Boxplots from selected proteins from the Olink Reveal panel |

**Table S1: Baseline characteristics of hyperkeratotic palmoplantar eczema (HPE), palmoplantar psoriasis (PP), and palmoplantar pustulosis (PPP)**

| **Baseline data** | **HPE**  (n = 14) | **PP**  (n = 10) | **PPP**  (n = 12) |
| --- | --- | --- | --- |
|  |  |  |  |
| **No anti-psoriatic,** topical treatment for 14 days, n (%) ^#^ | 12 (85.7) | 7 (70.0) | 10 (83.3) |
|  |  |  |  |
| **Anti-psoriatic,** topical treatment, n (%) ^#^ | 3 (21.4) | 3 (30.0) | 2 (16.7) |
|  |  |  |  |
| **Localisation of disease,** n (%) ^#^ |  |  |  |
| Only palm | 8 (57.1) | 2 (20.0) | 1 (8.3) |
| Only sole | 0 | 3 (30.0) | 3 (25.0) |
| Both palm and sole | 6 (42.9) | 5 (50.0) | 8 (66.7) |
|  |  |  |  |
| **Sample site, lesional,** n (%) ^#^ |  |  |  |
| Palm | 10 (71.4) | 3 (30.0) | 3 (25.0) |
| Sole | 4 (28.6) | 7 (70.0) | 9 (75.0) |
|  |  |  |  |
| **Ethnicity,** n (%) ^#^ |  |  |  |
| Caucasian | 13 (92.9) | 10 (100) | 12 (100) |
| Middle East | 1 (7.1) | 0 (0) | 0 (0) |
|  |  |  |  |
| **Genetic disposition for atopic dermatitis**, n (%) ^¶^ |  |  |  |
| Yes | 2 (14.3) | - | - |
| No | 8 (57.1) | - | - |
| Unknown | 4 (28.6) | - | - |
|  |  |  |  |
| **Concurrent plaque psoriasis,** n (%) ^#^ | - | 7 (70.0) | 6 (50.0) |
|  |  |  |  |
| **Psoriatic arthritis (PsA),** n (%) ^¶^ |  |  |  |
| Yes | - | 4 (40.0) | 0 (0) |
| No | - | 5 (50.0) | 12 (100) |
| Unknown | - | 1 (10.0) | 0 (0) |
|  |  |  |  |
| **NAPSI, total,** median (IQR) ^1, #^ |  | 2 (0.0 - 3.0) | 1 (0.0 - 3.5) |
| Nail matrix, n (%) |  |  |  |
| *Pitting* | - | 4 (40.0) | 3 (25.0) |
| *Leukonychia* | - | 1 (10.0) | 1 (8.3) |
| *Crumbling* | - | 0 (0) | 0 (0) |
| *Red spots* | - | 0 (0) | 0 (0) |
| Nail bed, n (%) | - |  |  |
| *Onycholysis* | - | 2 (20.0) | 2 (16.7) |
| *Subungual Hyperkeratosis* | - | 1 (10.0) | 2 (16.7) |
| *Oil drop* | - | 1 (10.0) | 1 (8.3) |
| *Splinters* | - | 2 (20.0) | 0 (0) |
|  |  |  |  |
| **Comorbidities,** n (%) ^2, ¶^ |  |  |  |
| Overweight | 3 (21.4) | 1 (10.0) | 1 (16.7) |
| Hypertension | 7 (50.0) | 4 (40.0) | 5 (41.7) |
| Hypercholesterolemia | 10 (71.4) | 1 (10.0) | 7 (58.3) |
| Diabetes mellitus 1 | 1 (7.1) | 0 (0) | 1 (8.3) |
| Diabetes mellitus 2 | 1 (7.1) | 0 (0) | 2 (16.7) |
| Thyroid disease | 2 (14.3) | 2 (20.0) | 1 (8.3) |
| Lung disease | 1 (7.1) | 1 (10.0) | 0 (0) |
| Kidney disease | 1 (7.1) | 0 (0) | 0 (0) |
| Liver disease | 1 (7.1) | 0 (0) | 0 (0) |
| Inflammatory Bowel Disease ^3^ | 1 (7.1) | 0 (0) | 0 (0) |
| Cancer | 2 (14.3) | 1 (10.0) | 0 (0) |
| Mental illness | 2 (14.3) | 1 (10.0) | 3 (25.0) |
|  |  |  |  |
| **Gene blood test,** n (%) |  |  |  |
| HLA-C*06:02 positive ^4^ | 2 (14.3) | 1 (10.0) | 4 (33.3) |

HPE: hyperkeratotic palmoplantar eczema, PP: palmoplantar psoriasis, PP: palmoplantar pustulosis, n: number, %: percent, IQR: interquartile range, PSA: psoriatic arthritis, NAPSI: nail psoriasis severity index, HLA-C: human leukocyte antigen belongs to the major histocompatibility complex (MHC) class I heavy chain receptors. Patient-reported variables: Diagnosis of psoriasis (patients told by a doctor), psoriatic arthritis, comorbidities, and education. Psoriatic arthritis is not reported in patients with HPE. A health care professional has investigated the other variables. Median with IQR (25th-75th percentiles) for non-normally distributed continuous variables. Number and frequency as percentages for categorical variables.

^¶^ Patient-reported questionnaires.

^#^ Health-care professional examination and questionnaires.

^1^ NAPSI: The absence of data due to nail polish meant that NAPSI could not be assessed. This applied to one patient with PP and two patients with PPP.

^2^ Comorbidities: One PP reported another skin disease, rosacea. Two HPE reported another skin disease, but the type was unknown.

^3^ Inflammatory Bowel Disease: Morbus Crohn or Colitis Ulcerosa.

^4^ HLA positive: Positive in one allele.

**Table S2: Participant demographics, disease severity, and treatment characteristics for longitudinal data of palmoplantar psoriasis (PP)**

| **Participant characteristics** | **PP, Baseline**  (n = 6) | **PP, Follow-up**  (n = 6) |
| --- | --- | --- |
|  |  |  |
| **Age,** years, median (IQR) | 62.5 (41.8 - 71.2) | 64 (43.0 - 72.2) |
| **Female,** n (%) | 5 (83.3) | - |
|  |  |  |
| **PPASI,** median (IQR) | 11 (4.4 - 13) | 9.3 (2.2 - 16.4) |
|  |  |  |
| **NAPSI,** median (IQR) ^1^ | 0 (0.0 - 6.0) | 2.5 (1.8 - 3.8) |
|  |  |  |
| **Untreated,** n (%) ^2^ | 3 (50) | 2 (33) |
|  |  |  |
| **Anti-psoriatic,** topical treatment, n (%) | 3 (50) | 2 (33) |
|  |  |  |
| **Anti-psoriatic,** systemic treatment, n (%) | 0 (0) | 2 (33) |
|  |  |  |

PP: palmoplantar psoriasis, N: number, %: percent, IQR: interquartile range, PPASI: palmoplantar psoriasis area and severity index, NAPSI: nail psoriasis severity index. Median with IQR (25th-75th percentiles) for non-normally distributed continuous variables. Number and frequency as percentages for categorical variables.

^1^ NAPSI: The absence of data due to nail polish meant that NAPSI could not be assessed. This applied to three visits. Range: minimum 0 - maximum 32.

^2^ Untreated definitions: No anti-psoriatic topical treatment for 14 days, no UV light treatment for one month, no anti-psoriatic systemic treatment for one month, and no advanced immunological systemic treatment for three months.

**Table S3: Key summary of variations in MSD S-PLEX and U-PLEX**

The following table summarizes the technical differences across the three parallel panel experiments.

| **Assay** | **S-PLEX** | **S-PLEX Panel** | **U-PLEX** |
| --- | --- | --- | --- |
| **Analytes** | IL-22 | IL-17A/TNFα | IL-23/IP-10 |
| **Coating** | Biotinylated Capture Ab + Reagent C1 | Biotinylated Capture Ab blend | Biotinylated Capture Ab coupled to linkers before coating |
| **Lower LOD** | Ultra-sensitive  – LOD: 2,2 fg/mL | Ultra-sensitive  – LOD: 1-29 fg/mL for TNFα and 6-130 fg/mL for IL-17A | Sensitive  – LOD: 1.4 pg/mL for IL-23 and 0.49 pg/mL for IP-10 |
| **Enhancement** | TURBO-TAG (27 °C) | TURBO-TAG (27 °C) | N/A (SULFO-TAG) |

MSD: Meso Scale Discovery, Ab: Anti-body, LOD: Lower limit of detection, were defined according to the manufacturer's specifications (MSD).

**Table S4: Proteins in Reactome pathways**

| **R-HSA-6798695 \| Neutrophil degranulation** | **R-HSA-168249 \| Innate Immune System** |
| --- | --- |
| ACAA1 | ACAA1 |
| ACP3 | ACP3 |
| ADAM8 | ADAM8 |
| ANXA2 | ANXA2 |
| ATAD3B | ATAD3B |
| CD63 | ATP6V0D1 |
| CEACAM3 | BAIAP2 |
| CEACAM8 | BPIFA2 |
| CKAP4 | CASP8 |
| CLEC12A | CD63 |
| CLEC4D | CEACAM3 |
| CLEC5A | CEACAM8 |
| CRACR2A | CKAP4 |
| CXCL1 | CLEC12A |
| ERP44 | CLEC4D |
| FCAR | CLEC5A |
| FGR | CRACR2A |
| GLIPR1 | CXCL1 |
| GSTP1 | DEFB103B |
| HMOX2 | DEFB103A |
| IRAG2 | ERP44 |
| LTA4H | FCAR |
| MME | FGR |
| PADI2 | GLIPR1 |
| PGM2 | GSTP1 |
| PRDX6 | HMOX2 |
| PSMD1 | HRAS |
| PTGES2 | IKBKG |
| RAB44 | IL18 |
| S100A12 | IL1B |
| TIMP2 | IRAG2 |
|  | LPO |
|  | LTA4H |
|  | MAPK9 |
|  | MAPKAPK2 |
|  | MME |
|  | NOS1 |
|  | NOS2 |
|  | PADI2 |
|  | PGLYRP3 |
|  | PGM2 |
|  | PRDX6 |
|  | PSMD1 |
|  | PTGES2 |
|  | PTPN11 |
|  | RAB44 |
|  | S100A12 |
|  | TAX1BP1 |
|  | TIMP2 |
|  | TLR1 |
|  | TRIM25 |

**Table S5: Mean NPX and changes from baseline to follow-up for the top five proteins in palmoplantar psoriasis (PP)**

| **Lesional baseline - Healed follow-up** | | | | | | | |
| --- | --- | --- | --- | --- | --- | --- | --- |
| **Assay** | **Mean NPX** | | **Difference in NPX** | | | | |
|  | **Lesional** | **Healed** | **Mean** | **SD** | **Median** | **logFC** | **% change** |
| CXCL10 | 4,982 | 0,119 | -4,863 | 2,859 | -5,602 | 0,024 | -97,618 |
| CXCL9 | 4,496 | -0,118 | -4,614 | 2,211 | -5,241 | -0,026 | -102,627 |
| MMP12 | 3,503 | -2,092 | -5,595 | 3,248 | -6,838 | -0,597 | -159,720 |
| PPL | 3,037 | -2,429 | -5,466 | 2,425 | -6,440 | -0,800 | -179,973 |
| PSME2 | 2,000 | -2,871 | -4,871 | 3,928 | -7,057 | -1,435 | -243,516 |
| **Lesional baseline - Persistent lesional follow-up** | | | | | | | |
| **Assay** | **Mean NPX** | | **Difference in NPX** | | | | |
|  | **Lesional** | **Healed** | **Mean** | **SD** | **Median** | **logFC** | **% change** |
| CXCL10 | 6,402 | 5,973 | -0,429 | 0,245 | -0,539 | 0,933 | -6,696 |
| CXCL9 | 5,337 | 5,106 | -0,231 | 0,065 | -0,231 | 0,957 | -4,333 |
| MMP12 | 4,515 | 4,427 | -0,088 | 1,172 | 0,505 | 0,981 | -1,942 |
| PPL | 4,437 | 3,580 | -0,857 | 0,516 | -0,901 | 0,807 | -19,312 |
| PSME2 | 3,811 | 3,570 | -0,240 | 0,946 | -0,741 | 0,937 | -6,310 |

NPX: normalized protein expression, SD: standard deviation, logFC: estimated log_2_ fold change between conditions, %: percent.

**Figure S1: Workflow of tape strip sampling, protein extraction, Olink Reveal analysis, next-generation sequencing (NGS), and data analysis**

Created in https://BioRender.com.


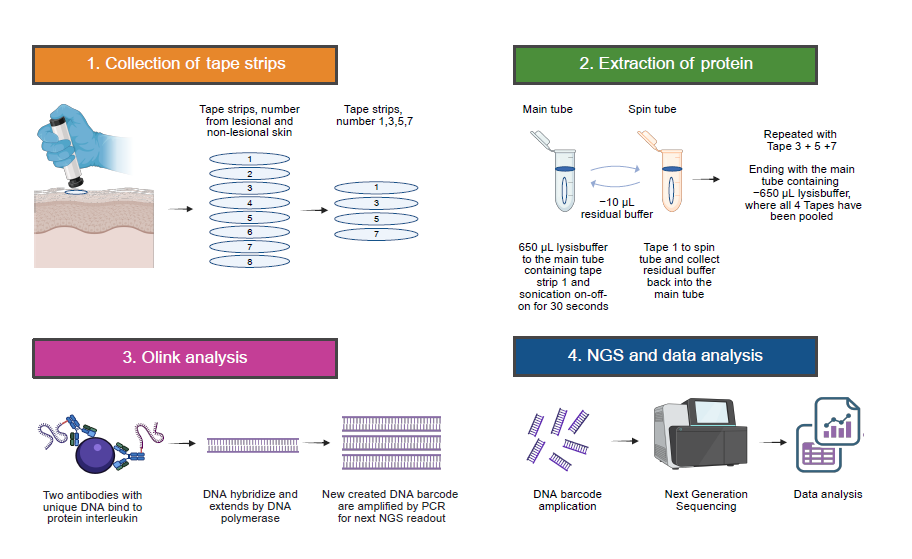


**Figure S2: Protein concentration was highest in lesional samples and in palmoplantar pustulosis (PPP)**

Protein levels (mg/mL) were quantified in samples from patients with hyperkeratotic palmoplantar eczema (HPE), palmoplantar psoriasis (PP), and palmoplantar pustulosis (PPP) and compared between lesional (L, red bars) and non-lesional (NL, turquoise bars) skin. Data are presented as medians with interquartile range (IQR).

**
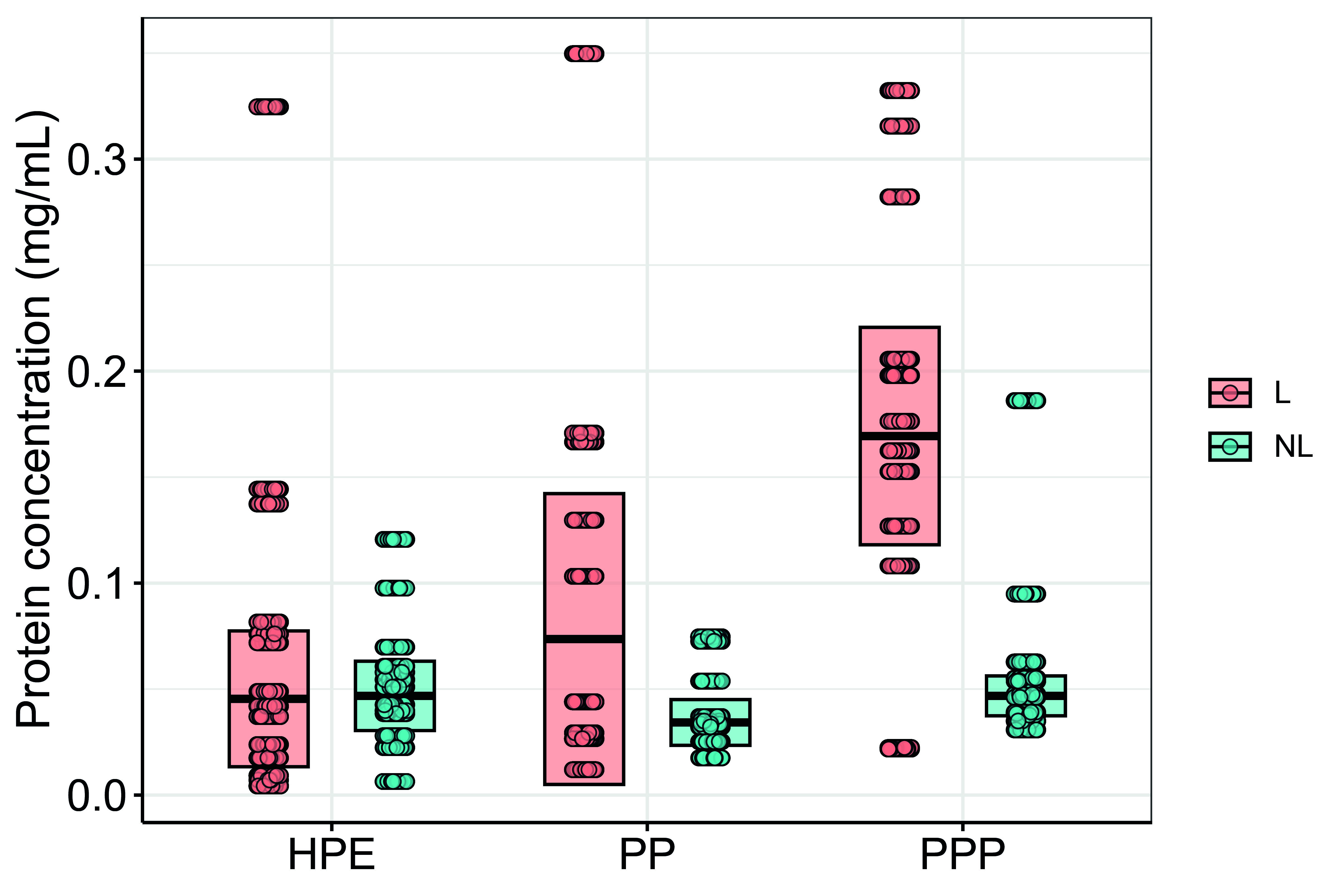
**

**Figure S3: Palmoplantar psoriasis (PP) and hyperkeratotic palmoplantar eczema (HPE) present similar proteomic signatures**

Volcano plot showing differential protein expression in lesional skin between PP-HPE. The x-axis represents log_2_ fold change, and the y-axis represents -log_10_ adjusted *p*-value. Vertical dashed lines indicate the fold-change threshold (foldchange = 2), and the horizontal dashed line indicates the significance threshold (adjusted *p*-value = 0.05). Each dot represents one protein; grey dots denote non-significant proteins. Selected proteins that were significantly differentially expressed in the other comparisons (PPP-HPE, PPP-PP) are labelled by their assay names. HPE: hyperkeratotic palmoplantar eczema, PP: palmoplantar psoriasis, PPP: palmoplantar pustulosis.

**
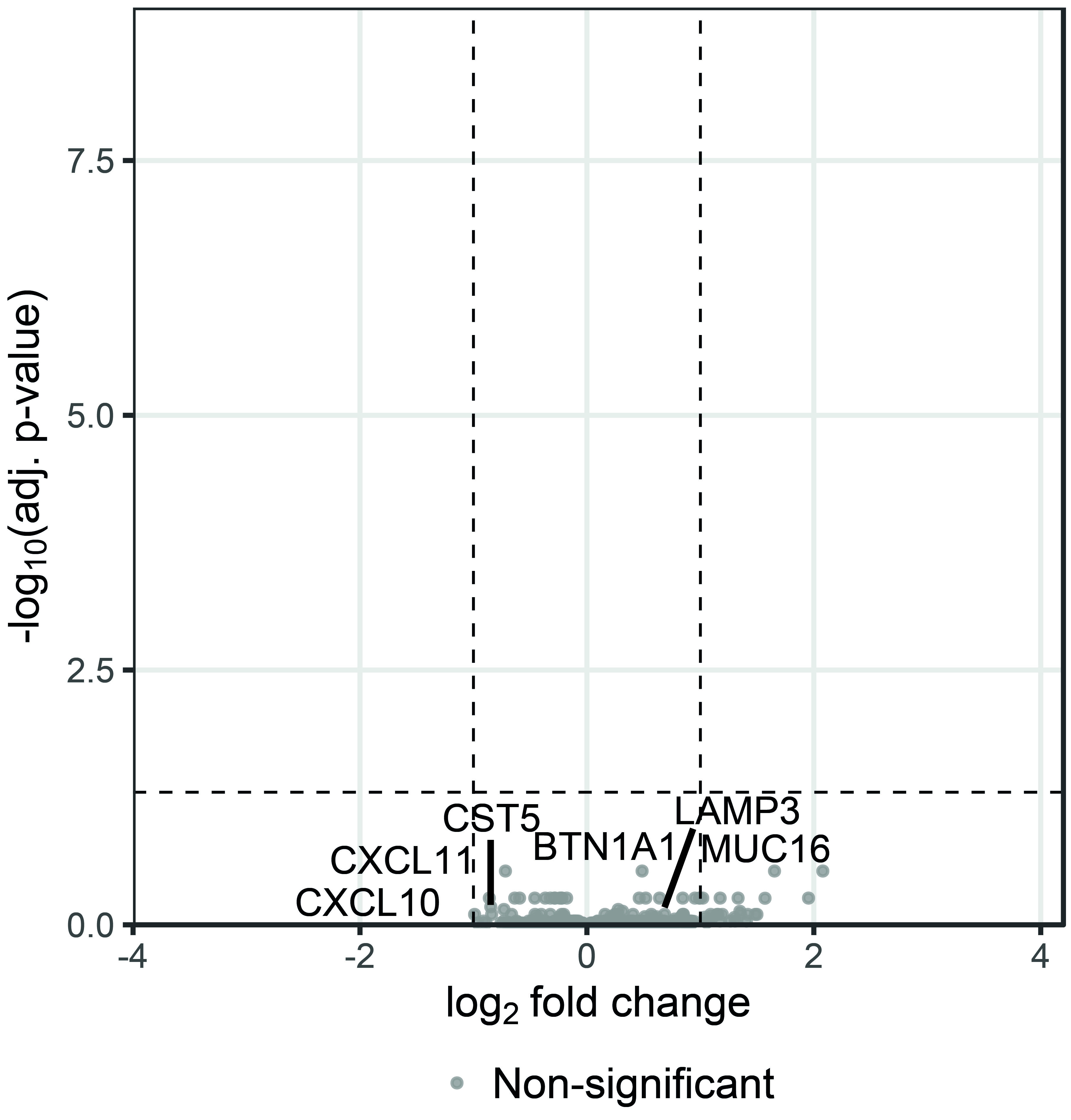
**

**Figure S4: Boxplots from selected proteins from the Olink Reveal panel**

Boxplots from selected proteins from the Olink Reveal panel that are relevant for downstream analysis. Y-axis: NPX values, x-axis: disease groups. HPE: hyperkeratotic palmoplantar eczema, PP: palmoplantar psoriasis, PPP: palmoplantar pustulosis. Red bars: lesional samples (L), turquoise bars: non-lesional samples (NL). Dotted line assay-specific LOD value.


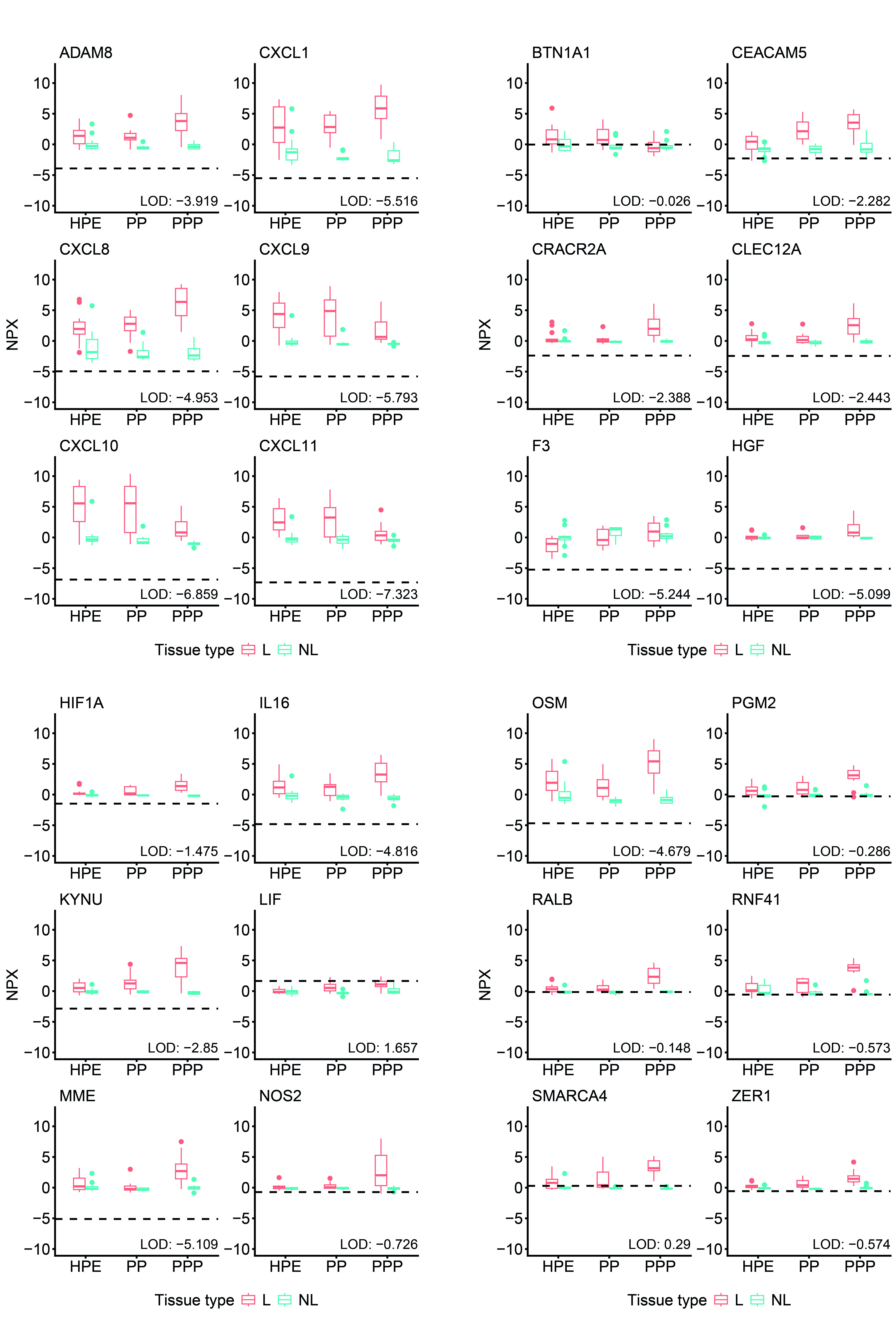

Supplement: Supplementary file 1 — Table S1: Baseline characteristics of hyperkeratotic palmoplantar eczema (HPE), palmoplantar psoriasis (PP) and palmoplantar pustulosis (PPP). Table S2: Participant demographics, disease severity and treatment characteristics for longitudinal data of palmoplantar psoriasis (PP). Table S3: Key summary of variations in MSD S‐PLEX and U‐PLEX. Table S4: Proteins in Reactome pathways. Table S5: Mean NPX and changes from baseline to follow‐up for the top five proteins in palmoplantar psoriasis (PP). Figure S1: Workflow of tape strip sampling, protein extraction, Olink Reveal analysis, next‐generation sequencing (NGS) and data analysis. Figure S2: Protein concentration was highest in lesional samples and in palmoplantar pustulosis (PPP). Figure S3: Palmoplantar psoriasis (PP) and hyperkeratotic palmoplantar eczema (HPE) present similar proteomic signatures. Figure S4: Boxplots from selected proteins from the Olink Reveal panel. [file EXD-35-e70318-s001.docx]
